# Laparoscopic approach to early gastric cancer in a patient with a prior history of open right hepatectomy: a case report

**DOI:** 10.1186/s40792-020-00847-4

**Published:** 2020-04-26

**Authors:** Ko Ikegame, Makoto Hikage, Satoshi Kamiya, Yutaka Tanizawa, Etsuro Bando, Masanori Terashima

**Affiliations:** Division of Gastric Surgery, Shizuoka Cancer Center, 1007 Shimonagakubo, Nagaizumi-cho, Sunto-gun, Shizuoka, 411-8777 Japan

**Keywords:** Laparoscopic gastrectomy, Gastric cancer, Prior history of hepatectomy

## Abstract

**Background:**

Laparoscopic gastrectomy is regarded a standard treatment procedure for early gastric cancer and is widely used in clinical practice. However, the feasibility of laparoscopic gastrectomy for patients with a prior history of open surgery, especially in the case of a complicated operation, remains unclear. Here, we report a laparoscopic gastrectomy case with a prior history of right hepatectomy.

**Case presentation:**

A 70-year-old man was diagnosed with early gastric cancers preceding a right hepatectomy for a solitary hepatocellular carcinoma at risk of rupture. An additional gastrectomy, after non-curative endoscopic submucosal dissection, was planned after the hepatectomy. Extensive adhesions were found around the liver. Rigid adherence of the duodenum to the adjacent hepatoduodenal ligament had formed. In addition, identification of the hepatic artery was difficult due to stiffening of the mesentery. Peeling off the adhesions from the ventral side of the duodenum revealed the supra-pyloric vessels and enabled us to transect the duodenum safely. Further, exposing the proper hepatic artery via the dorsal side of the mesentery and subsequent supra-pancreatic dissection on the outermost layer allowed effective identification of the right gastric artery. The postoperative course was uneventful.

**Conclusions:**

We successfully performed total laparoscopic distal gastrectomy on a patient with a prior history of major hepatectomy.

## Background

Laparoscopic gastrectomy is regarded a standard treatment for early gastric cancer and is being widely used in clinical practice. Laparoscopic approach is increasingly being used for patients with a history of abdominal surgery [1]. However, it is unclear whether laparoscopic gastrectomy can be performed in patients with a history of major abdominal surgery. So far, there have been no reports of laparoscopic gastrectomy in patients with a history of open hepatectomy. Herein, we report a patient with a prior history of right hepatectomy who underwent laparoscopic distal gastrectomy.

## Case presentation

A 70-year-old man visited our hospital for treatment of solitary hepatocellular carcinoma in segments 5 and 6. Examination revealed four lesions: two indicative of early gastric cancer and two of suspected adenocarcinoma. The first lesion was a type 0-IIa well-differentiated tubular adenocarcinoma with a diameter of 16 mm. The second was a type 0-IIc well-differentiated tubular adenocarcinoma with a diameter of 10 mm. The third was a type 0-IIc lesion of suspected adenocarcinoma with a diameter of 10 mm. The last lesion was a type 0-IIc lesion of suspected adenocarcinoma with a diameter of 3 mm. The diagnosis of all cancer lesions and the two suspicious of adenocarcinoma were an absolute indication for endoscopic submucosal dissection (ESD). Before undergoing hepatic surgery, percutaneous portal embolization (PTPE) was performed to increase the future remnant liver volume (FRLV). FRLV was hypertrophied from 334 to 563 ml after PTPE, and the resection rate was decreased from 963 ml (74.3%) to 1144 ml (67.0%). Then, a right hepatectomy was performed preceding gastric cancer treatment. The Pringle maneuver was performed during liver transection. An anti-adhesion membrane was spread out on the right subphrenic space, inferior vena cava, cut liver stump, hepatoduodenal ligament, and under the wound. Endoscopic submucosal dissection was performed 2 months after the hepatectomy, and the first lesion was diagnosed as eCuraC-2 due to submucosal tumor invasion. Neither swollen lymph nodes nor distant metastases appeared in preoperative imaging diagnoses. While considering surgical and oncological safety, an additional laparoscopic gastrectomy was planned upon the patient’s request and was to be carried out 4 months after the hepatectomy.

**Fig. 1:**
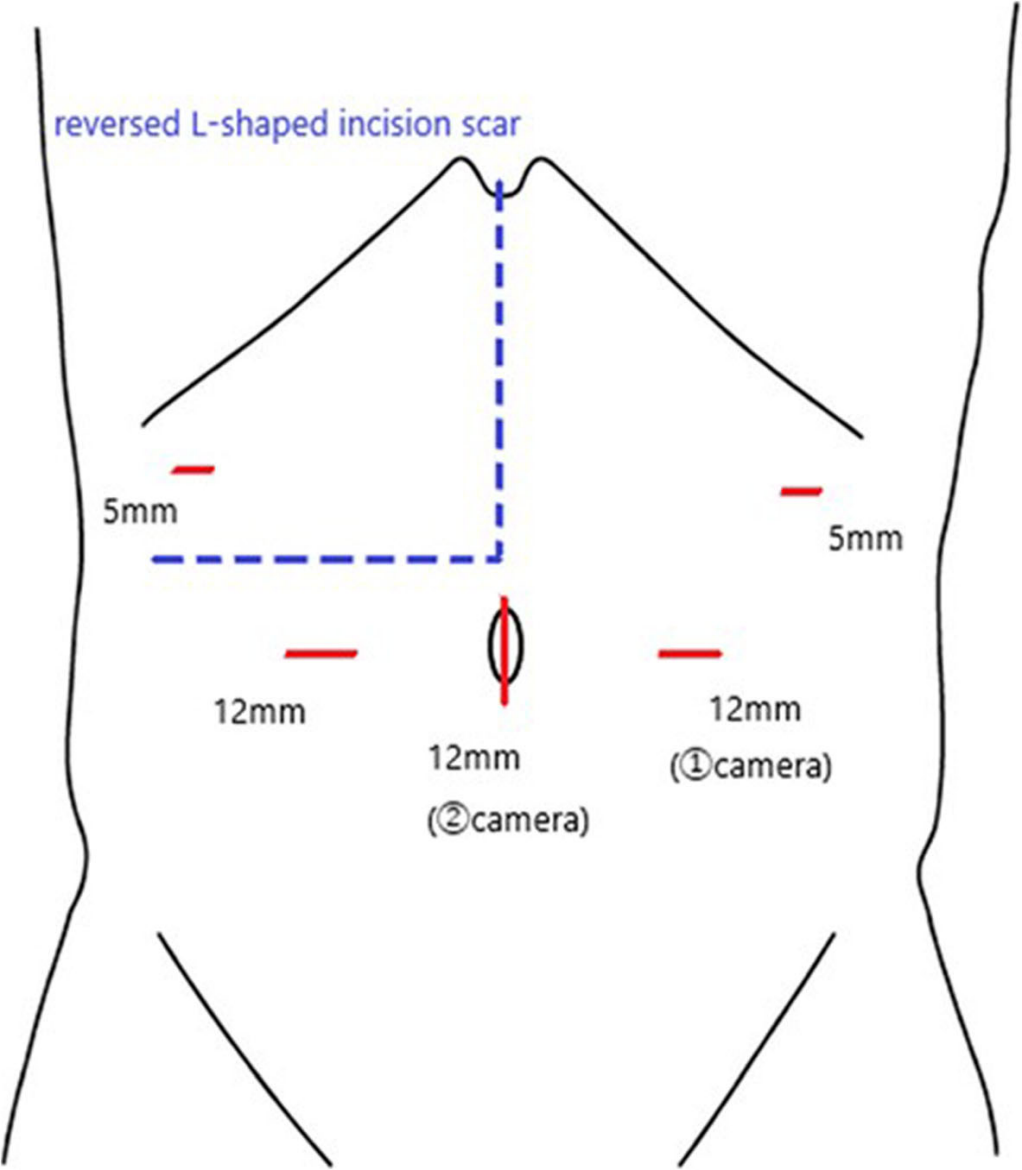
The port location. The 1st port was inserted away from the reversed L-shaped incision scar

Extensive adhesions were found, especially around the resected margin of the liver. Though the enlarged remnant left hepatic lobe was an obstacle for surgery, lifting up the liver by the cut round ligament effectively created a better surgical field. Laparoscopic adhesiotomy was started from adhesion-free space on the left abdomen (Fig. 1).

**Fig. 2:**
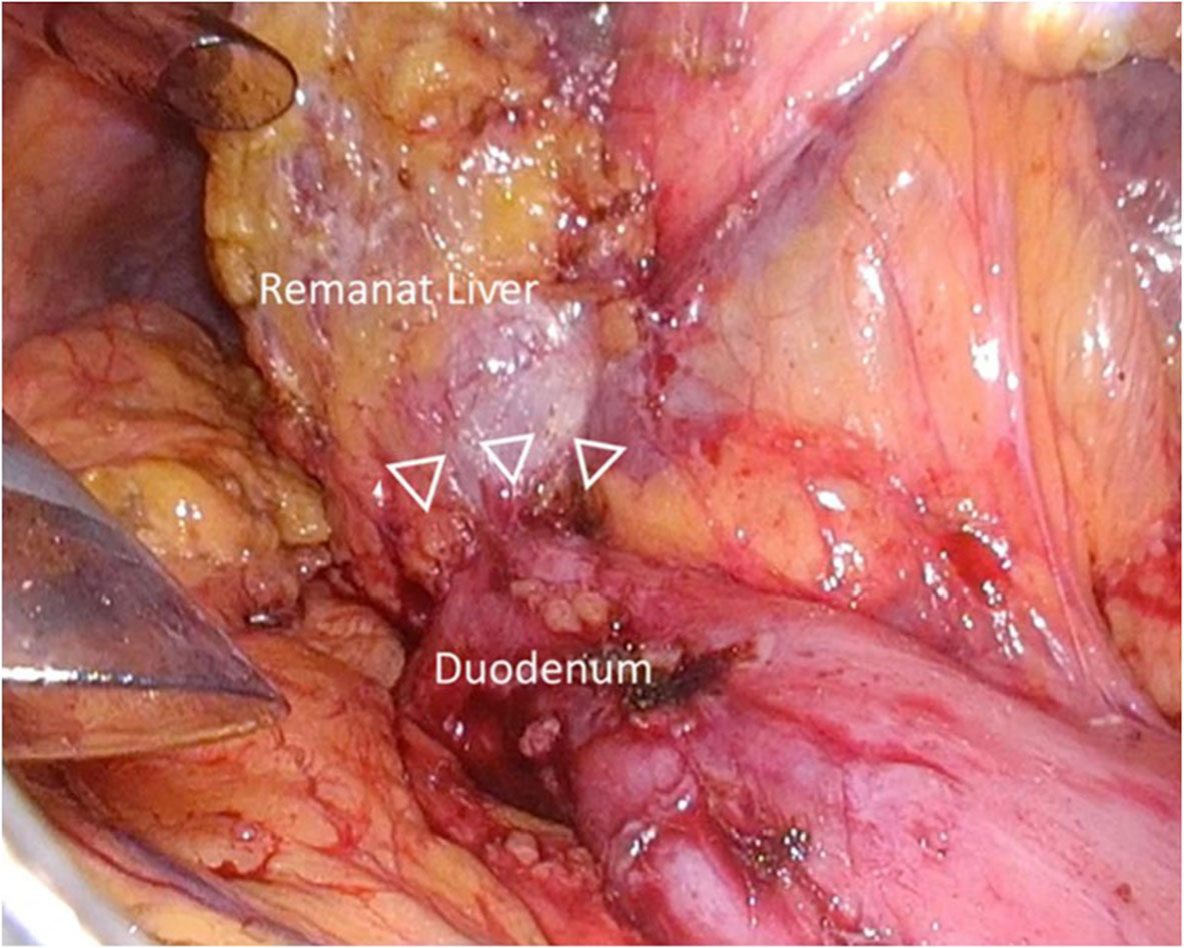
Rigid adherence of the duodenal bulb to the adjacent hepatoduodenal ligament was formed

**Fig. 3:**
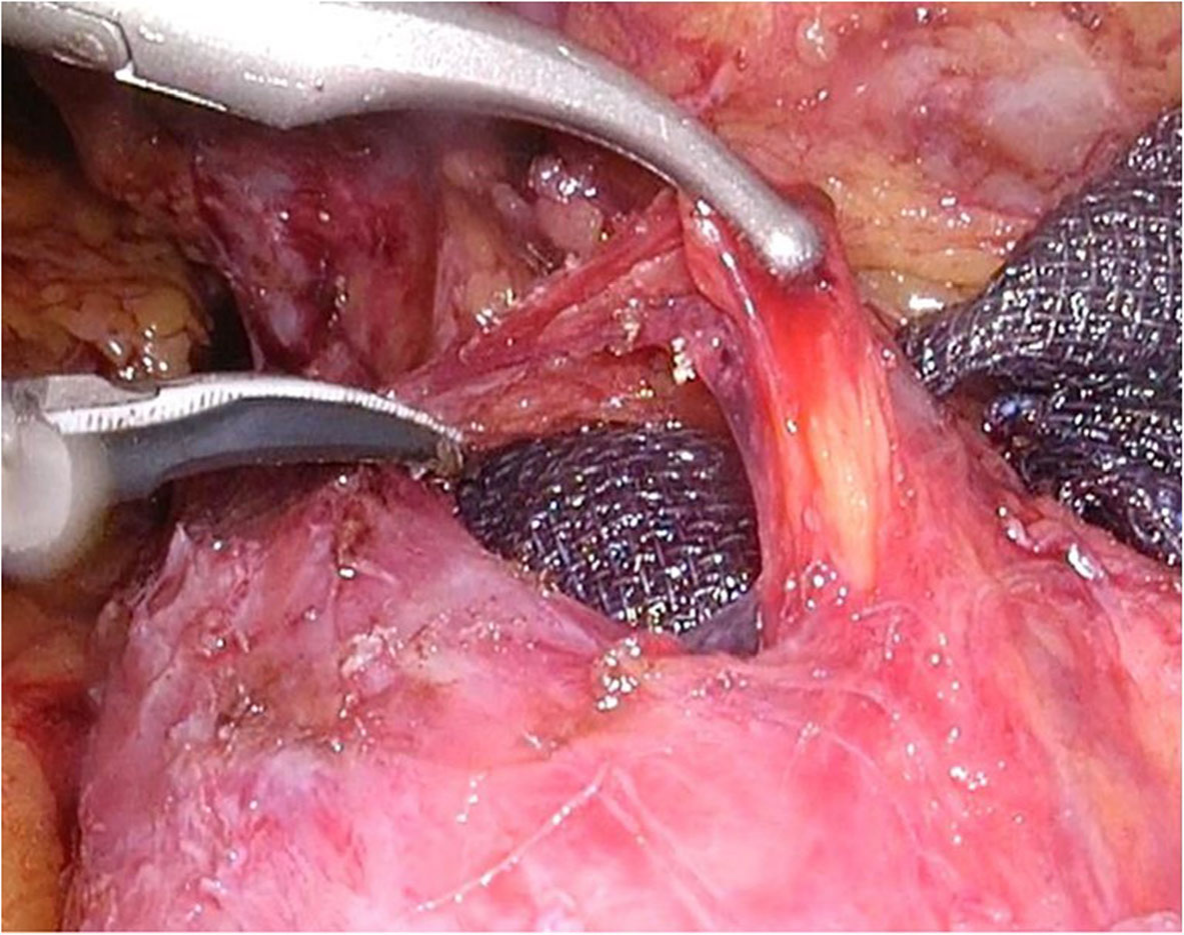
Breaking up adhesions from the ventral side of the duodenum revealed the location of the duodenal arteries

**Fig. 4:**
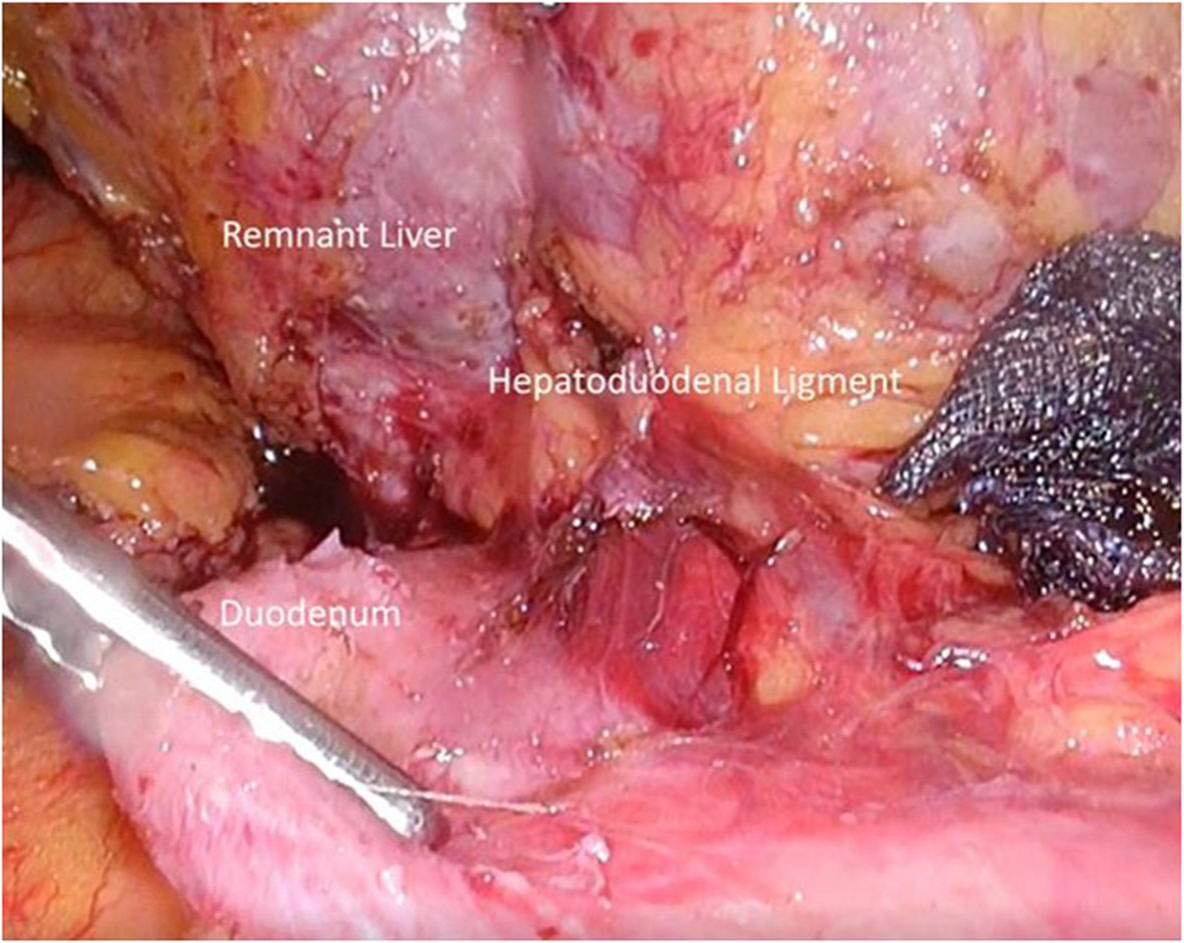
The right gastric artery could not be identified due to stiffening of the hepatoduodenal ligament

**Fig. 5:**
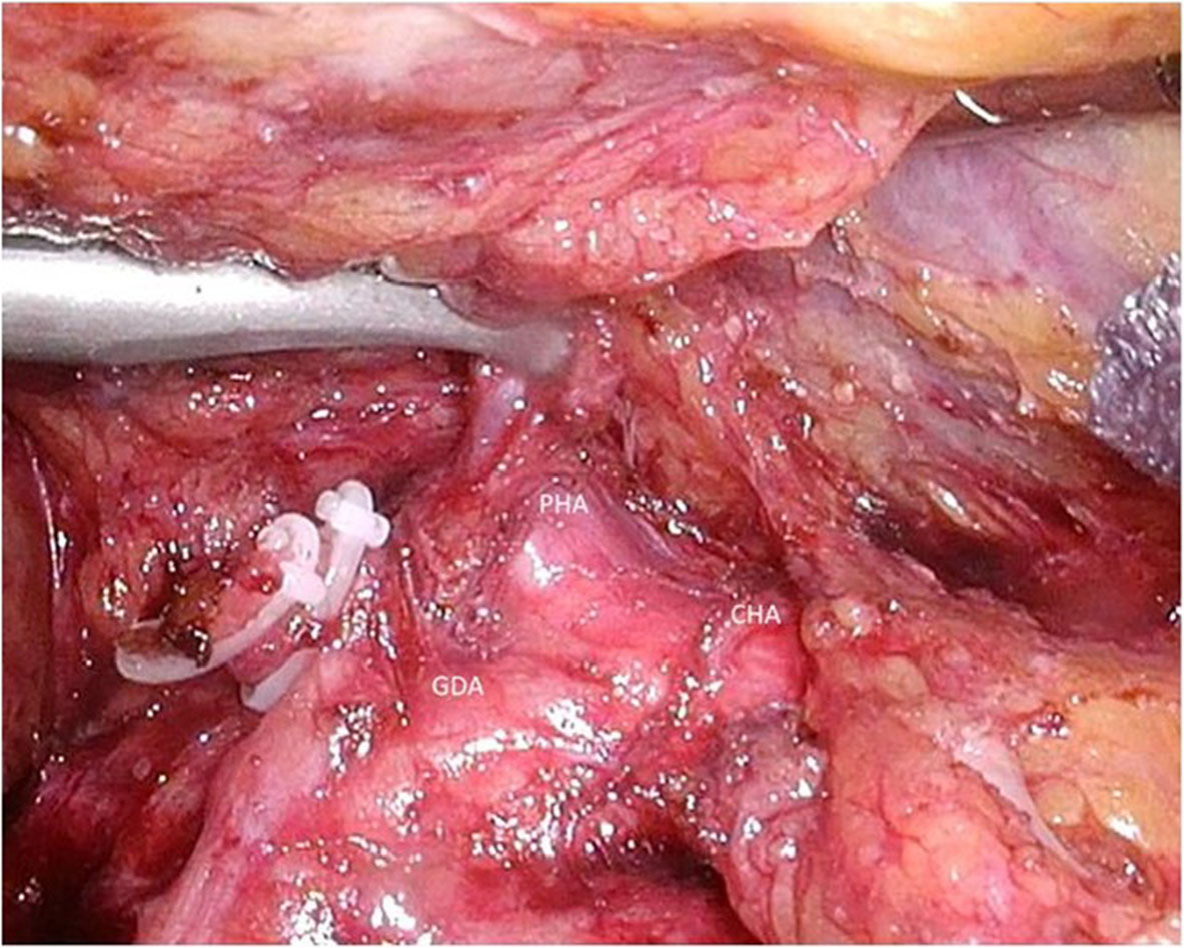
After exposing the PHA by dissection of consecutive layers, both the GDA and the CHA, a pedicle of the RGA was divided safely. PHA, proper hepatic artery; GDA, gastroduodenal artery; CHA, common hepatic artery; RGA, right gastric artery


The transverse colon was adhered to the cut stump of remnant liver and obstructed vision of the duodenum. Sufficient mobilization of the transverse porcolon via the fusion fascia on the transverse mesocolon revealed a section of the duodenum displaced by adherence to the portal hepatis (Additional video 1). Rigid adherence of the duodenal bulb to the hepatoduodenal ligament due to previous surgery was found (Fig. 2). We carefully peeled off the adhesions from the ventral side of the duodenum, which enabled us to visualize the localization of the supra-pyloric vessels (Fig. 3). It was challenging to identify the right gastric artery via the ventral side because of the stiffened area around the hepatoduodenal ligament (Fig. 4). We then exposed both the gastroduodenal and common hepatic arteries via dissection on the outermost layer after transection of the duodenum. Localization of the proper hepatic artery was revealed by dissection of the next consecutive layer, and a pedicle of the right gastric artery was divided safely (Fig. 5).

**Additional file 1: Video 1.** Sufficient mobilization of the transverse porcolon via the fusion fascia on the transverse mesocolon revealed a section of the duodenum displaced by adherence to the portal hepatis.


**Fig. 6:**
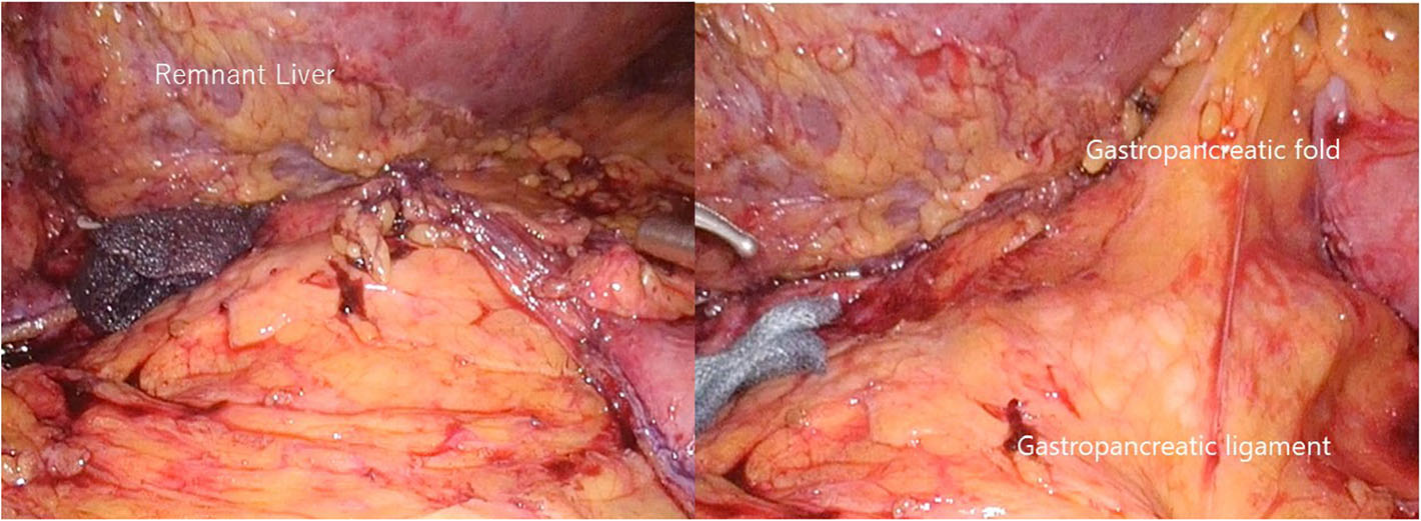
Adjusting the traction on the gastropancreatic ligament was necessary for managing a limited surgical field by the enlarged remnant liver

Consecutively, supra-pancreatic lymph nodes were dissected to accomplish the D1+ lymph node dissection. In brief, the lymph nodes along the common hepatic artery (station 8a) were removed en bloc just anterior to the portal vein. The lymph nodes located at both sides of the celiac artery (station 9) were dissected, and the root of the left gastric artery was clipped and divided. Adjusting the traction on the gastropancreatic ligament was necessary due to a limited surgical field from the enlarged left lobe of the liver (Fig. 6). Total laparoscopic distal gastrectomy with D1+ dissection followed by Roux-en-Y reconstruction was completed. The operative time was 306 min, and the estimated blood loss was 100 ml. The postoperative course was uneventful, and the patient was discharged on postoperative day 9.

## Discussion

We completed total laparoscopic distal gastrectomy for an early gastric cancer patient with a prior history of open right hepatectomy. Laparoscopic gastrectomy is widely recognized as a safe and effective procedure comparable with open gastrectomy [2]. However, for a patient with prior upper abdominal open surgery, feasibility of the laparoscopic approach for gastric cancer remains unclear. Some reports have mentioned that abdominal surgical history alone is not a contraindication for laparoscopic gastrectomy, providing an experienced surgeon carries out the surgery [3, 4]. However, in these reports, most previous surgeries were minor, such as cholecystectomy and appendectomy. Therefore, the results of these reports may not be applicable to the present case, with a history of major upper abdominal surgery. To the best of our knowledge, this is the first report of total laparoscopic gastrectomy for a gastric cancer patient with a prior history of major hepatectomy. In almost all patients with a history of hepatobiliary surgery, strong adhesions around the hepatic hilum are noted [5]. These adhesions displace the position of the intestinal and blood vessels. Due to the invasive nature of a major hepatectomy, we have preferentially performed many Pringle maneuvers, which could strengthen the adhesions around the hepatoduodenal ligament. In this case, multiple Pringle maneuvers and the remnant right-side stump of the liver have affected the condition around the duodenum. Dissection of the consecutive layer from a less-affected site allowed for a safe procedure. Laparoscopic surgery allows for recognition of ill-defined borders of adhesion by the magnification of visual scale. Moreover, advanced surgical tools, such as fine forceps and sophisticated energy devices, make laparoscopic surgery safer. In this case, we performed an additional gastrectomy at 4 months after hepatectomy. Some literature referred to the relationship between a waiting duration and the hardness of adhesion in repeat surgeries, while others suggested that this made the repeated surgery easier due to its loosening [6, 7]. Furthermore, we have previously published that a wait time of fewer than 6 months may be acceptable in cStage I gastric cancer [8]. Performing surgery within this time may minimize associated surgical risks that arise from an adhesion.

However, it is difficult to show the benefit of selecting a laparoscopic approach for a patient with a history of major hepatobiliary surgery based only on a single case. In addition, a long operative time was required in comparison with common laparoscopic gastrectomy [2]. Laparoscopic approaches should be selected only for cases without oncological complications. This challenging procedure is only advisable when all parties have modestly considered safety and oncological radicality.

## Conclusion

We successfully performed total laparoscopic distal gastrectomy in a patient with a history of major hepatectomy.

## Data Availability

This case report does not have a dataset. All related data are included within the article.

## Abbreviations

PTPE: Percutaneous portal embolization
FRLV: Future remnant liver volume
